# mTOR at the Transmitting and Receiving Ends in Tumor Immunity

**DOI:** 10.3389/fimmu.2018.00578

**Published:** 2018-03-27

**Authors:** Yakir Guri, Thierry M. Nordmann, Jason Roszik

**Affiliations:** ^1^Biozentrum, University of Basel, Basel, Switzerland; ^2^Department of Dermatology, University Hospital Zurich, Zurich, Switzerland; ^3^Department of Melanoma Medical Oncology, The University of Texas MD Anderson Cancer Center, Houston, TX, United States; ^4^Department of Genomic Medicine, The University of Texas MD Anderson Cancer Center, Houston, TX, United States

**Keywords:** signaling, tumorigenesis, metabolism, immunity, immunotherapy, rapamycin

## Abstract

Cancer is a complex disease and a leading cause of death worldwide. Immunity is critical for cancer control. Cancer cells exhibit high mutational rates and therefore altered self or neo-antigens, eliciting an immune response to promote tumor eradication. Failure to mount a proper immune response leads to cancer progression. mTOR signaling controls cellular metabolism, immune cell differentiation, and effector function. Deregulated mTOR signaling in cancer cells modulates the tumor microenvironment, thereby affecting tumor immunity and possibly promoting carcinogenesis.

## Introduction

Tumor bulk is a mass containing heterogeneous cell populations including malignant cancer cells, non-malignant cells, and supporting stroma (1). In addition to tumor cells, non-malignant cells and the supporting stroma play a dynamic and possibly tumor promoting role (2). Non-malignant cells in the tumor microenvironment include cells of the lymphoid and myeloid immune system (3). The supporting stroma is largely composed of cancer-associated fibroblasts (CAFs), vascular and lymphatic endothelial cells, and pericytes. Cells within the tumor “communicate” by secretion of various factors to the tumor microenvironment, including matrix remodeling enzymes, cytokines, chemokines, growth factors, and metabolites (4, 5). This interplay between malignant, non-malignant, and stromal cells has functional consequences on tumor progression.

**Figure 1:**
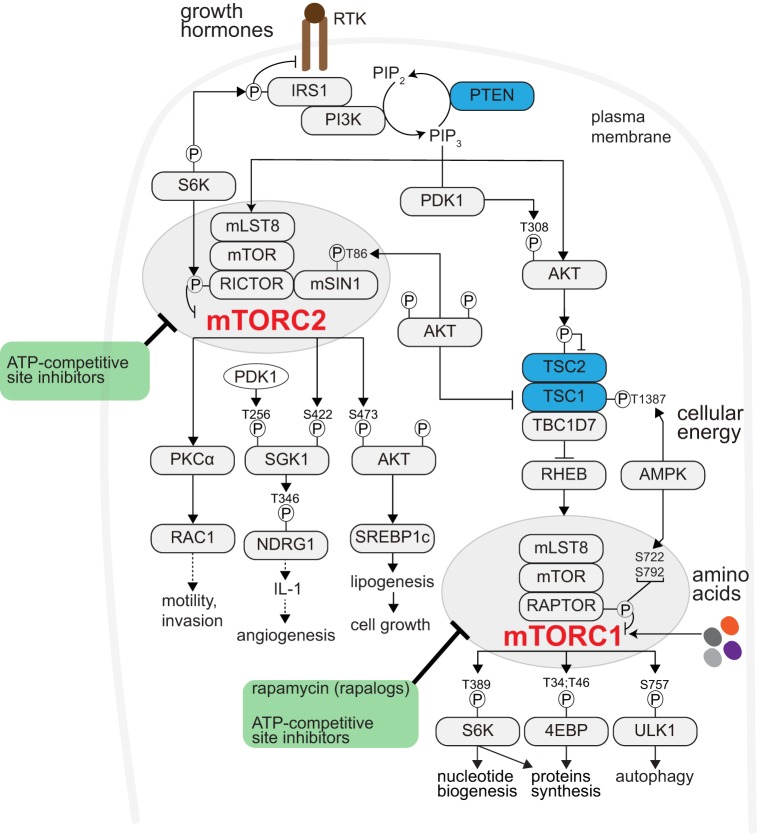
mTOR signaling promotes anabolism. Receptor Tyrosine Kinases (RTKs)- Phosphatidyl-Inositol-4,5-bisphosphate 3-Kinase (PI3K) activated by growth factor (like insulin). PI3K generates phosphatidylinositol-3,4,5-trisphosphate (PIP3) from the membrane phospholipid phosphatidylinositol-4,5-bisphosphate (PIP2). Phosphatase and Tensin Homolog Deleted on Chromosome 10 (PTEN) counteracts PI3K activity (restoring PIP3 to PIP2). PIP3 recruits to the plasma membrane and activates phosphoinositide-dependent kinase 1 (PDK1) and AKT. PDK1 phosphorylates and activates AKT (pAKT-Thr308). pAKT-Thr308 phosphorylates and inhibits the TSC complex. The TSC complex, composed of tuberous sclerosis complex 1 (TSC1) and TSC2 and TRE2-BUB2-CDC16 domain family member 7 (TBC1D7), activates the lysosomal RAS homolog enriched in brain (RHEB). RHEB interacts with and activates mTORC1. mTORC1 comprises mTOR, mammalian lethal with sec-13 protein 8 (mLST8), and regulatory-associated protein of mammalian target of rapamycin (RAPTOR). mTORC1 can also be activated by nutrients (such as amino acids). Cellular energy status also regulates mTORC1 through AMPK-mediated TSC or RAPTOR phosphorylation. mTORC1 promotes anabolism, among others, through ribosomal protein S6 kinase (S6K), eukaryotic translation initiation factor 4E (eIF4E)-binding protein 1 (4EBP1), and blocks cellular catabolism through Unc-51-like kinase 1 (ULK1). Through S6K-mediated IRS1 phosphorylation, mTORC1 negatively regulates mTORC2-AKT signaling. Rapamycin and its analogs (so-called rapalogues) acutely inhibit mTORC1 allosterically. The ATP-site competitive inhibitor(s) potently block both mTORC1 and mTORC2 signaling. mTORC2 is also activated by RTKs, and consists of mTOR, mLST8, mammalian stress-activated map kinase-interacting protein 1 (mSIN1), and rapamycin-insensitive companion of mTOR (RICTOR). mTORC2 regulates the AGC kinase family members AKT, serum/glucocorticoid-regulated kinase (SGK), and protein kinase C (PKC). Prolonged rapamycin administration may block mTORC2 activity.

Target Of Rapamycin (TOR) is an evolutionarily conserved serine/threonine protein kinase. TOR controls cellular metabolism and growth and functions in two complexes: TOR Complex 1 (TORC1) and TORC2 (6, 7) (Figure 1). Mammalian TORC1 (mTORC1) comprises mTOR, mammalian lethal with sec-13 protein 8 (mLST8), and regulatory-associated protein of mammalian target of rapamycin (RAPTOR). mTORC1 is activated by growth factors, nutrients (amino acids), and cellular energy (8, 9), and is allosterically inhibited by rapamycin (10). Various growth factors regulate mTORC1 *via* a heterotrimeric tuberous sclerosis complex (TSC) complex. Growth factors bind receptor tyrosine kinases (RTKs) and activate Phosphatidylinositol-4,5-Bisphosphate 3-Kinase (PI3K), which generates Phosphatidylinositol-3,4,5-Trisphosphate (PIP3) (11). PI3K activity is counteracted by the tumor suppressor, phosphatase, and Tensin Homolog Deleted on Chromosome 10 (PTEN). mTORC1 promotes anabolic processes, such as protein and nucleotide synthesis and inhibits catabolic processes, such as autophagy (12–14). mTORC2 contains mTOR, mLST8, mammalian stress-activated map kinase-interacting protein 1 (mSIN1), and Rapamycin-Insensitive Companion of mTOR (RICTOR), and is activated by growth factors in association with ribosomes (15) (Figure 1). mTORC1 and mTORC2 are frequently activated in human cancers and, as discussed below, reported to modulate the tumor microenvironment or respond to its changes.

## Cancer Cell-Intrinsic mTOR Activation Modulating the Tumor Microenvironment

Oncogenic mutations drive tumorigenesis by activating various growth controlling signaling pathways (16). The PI3K–mTOR–AKT signaling pathway is activated in the majority of tumors, due to upstream oncogenic mutation(s). Alternatively, parallel growth controlling (oncogenic) pathways, such as the MEK–ERK, may also activate PI3K–mTOR–AKT signaling (12). Either way, PI3K–mTOR–AKT activation promotes cell growth and proliferation (Figure 1). In addition to the cell-intrinsic growth-promoting effect, PI3K–mTOR–AKT activation appears to alter the tumor microenvironment.

## T Lymphocytes

T cells play a critical role in adaptive and innate immunity. Antigen recognition and adaptive immunity involves, among others, CD4 + and CD8 + T cells. While tumor eradication is largely mediated by cytotoxic CD8 + T lymphocytes (CTL), CD4 + T cells are critical in regulating and propagating the immune response, hence referred to as T helper cells (Th) (17). In solid tumors, the extent of T-cell infiltration is an important prognostic determinate. Increased CD4 + and CD8 + T-cell levels are associated with an improved clinical outcome (18). In colorectal tumors, increased density of T cells (i.e., Th1 adaptive immunity) correlated with reduced tumor recurrence, and provided a better prognostic tool than conventional histopathological methods (19). Conversely, tumors with a higher density of immune-suppressive cells (such T regulatory cells, as discussed below) exhibit a worse prognosis, in colorectal (19) and other tumor types (20). Thus, adaptive immunity plays a critical role in tumor progression and prognosis.

Various cytokines and chemokines attract immune cells to the site of inflammation (21). In addition to cytokines and chemokines, also metabolites in the tumor microenvironment (some of which are secreted by cancer cells) activate immune cells (22). Non-Alcoholic Fatty Liver Disease (NAFLD) is a metabolic disorder and a risk factor for hepatocellular carcinoma (HCC) (23). In NAFLD, increased linoleic acid levels disrupt adaptive immunity, specifically by depleting CD4 + T cells, which in turn promotes HCC (24). These data indicate that a metabolite accumulating in the tumor microenvironment may affect neighboring T cells, disturb their function, and promote cancer. It is not fully understood what regulates linoleic acid accumulation, but hepatic fatty acid (FA) synthesis (including linoleic acid) is controlled by mTORC2 (25). Importantly, constitutively active hepatic mTORC2 signaling is oncogenic and promotes HCC (26), and is particularly important in case of NAFLD to HCC transition (27). Thus, it is likely that mTORC2-mediated FA (and perhaps lipid) synthesis in cancer cells modulates immunity.

mTORC2 mediates various cellular processes *via* AGC kinase family members AKT, serum/glucocorticoid-regulated kinase (SGK), and protein kinase C (PKC) (28, 29) (Figure 1). In a mammary gland tumor model, *Rictor* deletion disrupted secondary mammary ductal branching, cell motility, and survival. This effect was mediated by PKCα-Rac1, but not AKT (30), suggesting an AKT-independent role of mTORC2 in motility and metastasis. mTORC2 phosphorylates and activates AKT (pAKT-Ser473). Melanoma with increased pAKT-Ser473 correlated with reduced T-cell infiltration, possibly due to increased secretion of inhibitory cytokines by cancer cells, and exhibit resistance to immune checkpoint inhibitors (31). The mTORC2 target SGK is frequently expressed in tumors (32). In gastric tumors, increased expression of the SGK1 target, NDRG1, is suggested to stimulate IL-1 expression and promote angiogenesis (33). Taken together, these data suggest that increased PI3K–mTORC2–AKT signaling in cancer cells may affect T cells and thereby tumorigenesis. It is possible that other immune cells in the tumor microenvironment are also modulated by PI3K–mTORC2–AKT, as described further below.

## Regulatory T Cells (Tregs)

Regulatory T cells suppress inflammation and are detrimental in tumor immunity. Genetic and pharmacological (rapamycin) abrogation of mTOR signaling induce Treg expansion *via* Foxp3 expression (34, 35). Furthermore, Treg-specific conditional TSC deletion in mice (constitutively active mTORC1) propelled Treg differentiation and a strong effector-like phenotype, reversed by S6K1 knockdown (36), suggesting that mTORC1 is an important checkpoint in Treg homeostasis.

Programmed Death 1 (PD-1) and Cytotoxic T-Lymphocyte-associated Antigen 4 (CTLA-4) immune checkpoints negatively regulate T-cell immune function. Immune suppression in the tumor microenvironment through PD-1 or CTLA-4 occurs in various tumors, and immune checkpoint inhibitors (anti-PD-1, anti-PD-L1, or anti-CTLA-4) amplify antitumor T-cell response (37). The surface protein PD-L1 is widely expressed in various tumors. PD-L1 binds to either the T-cell-expressed PD-1 or CD80 receptors thereby inhibiting their effector responses. PD-L1 and PD-1 interaction induces differentiation of naïve CD4 + T cells into Tregs, leading to an immune suppressive environment. In addition to inhibiting T-cell effector function, cancer cell-intrinsic PD-1 expression may promote tumor growth (38). Thus, PD-1 axis has a twofold effect in tumorigenesis: first by inhibiting cancer cell clearance by T cells, and second, promoting cancer cell growth. In a lung carcinoma mouse model, mTORC1 increased PD-L1 expression, allowing cancer cells to escape killing by immune cells (39, 40). Within the tumor, PD-L1 seems to be enriched in Tumor Initiating Cells (TICs) (also referred to as Cancer Stem Cells) (41–43). TICs are tumor cells with self-renewal capacity and considered to be more resistant to targeted cancer therapies. In syngeneic ovarian mouse model experiments, PD-L1 appeared to control the expression of canonical “stemness” genes, such as *Oct4* and *Nanog* (44, 45). PD-L1 expression correlated with mTOR activation in human lung adenocarcinomas and squamous cell carcinomas (39), suggesting that oncogenic AKT-mTOR activation promotes immune escape through PD-L1 upregulation. Furthermore, anti-PD-1 therapy inhibited human melanoma xenograft growth and reduced S6 phosphorylation, suggesting that PD-1 in tumor cells activates mTORC1. Importantly, cells expressing high levels of PD-L1 appear to be more sensitive to the mTORC1 inhibitor rapamycin, further suggesting that some of the PD-L1 growth-controlling mechanisms are *via* mTOR signaling. Collectively, these data suggest a functional relationship between mTOR signaling, PD-L1 expression, and resistance to targeted therapies (i.e., TICs). However, the mechanism(s) by which mTORC1 signaling regulates PD-L1 expression remains to be elucidated. We note that in addition to Treg and Th1, other T-cell subsets, such as Th17, may be involved in cancer immune response.

## Tumor-Associated Macrophages (TAMs)

Tumor-associated macrophages originate from expansion of tissue-resident macrophages or are recruited to tumor site (by chemotactic factors), and are present at multiple stages of tumor progression (2). Macrophages are not a homogenous population and can be subdivided into M1 and M2. M1 macrophages produce Th1 cytokines, promoting phagocyte-dependent inflammation and thereby an antitumor response. M2 macrophages enforce antibody response, but inhibit several phagocytic functions, therefore seemingly enabling a growth-tolerant tumor microenvironment. TAMs predominantly exhibit M2 phenotypes, therefore considered tumor-promoting. Several factors can promote polarization of TAMs to M2 during cancer progression, including IL-4, IL-10, TGF-β, and M-CSF (46). TAMs promote tumorigenesis by modulating lymph- and angiogenesis (47), but more recently, TAMs were shown to express PD-1. The presence of TAM expressing PD-1 steadily increases with cancer progression and results in an overall reduction in cancer cell phagocytosis (48). Because macrophages activation and function is, at least in part, controlled by PI3K–mTOR–AKT (49), it would be valuable to examine whether the observed reduction in phagocytosis is related to mTOR signaling. Furthermore, mTOR regulates macrophage polarization (50), and M1 and M2 macrophages exhibit dependency on distinct metabolic pathways. While M1 macrophages upregulate glycolysis and lipogenesis, M2 macrophages upregulate beta-oxidation. This is important because metabolic shifts are coupled to macrophage function (51, 52). For instance, IL-4 activate AKT and thereby inducing M2 gene transcription, possibly *via* ACLY expression and regulation of histone acetylation (53), indicating that mTOR signaling couple metabolic inputs to modulate immune response. Moreover, PI3K–AKT appears to recruit immune-suppressive monocytes to tumors *via* monocyte chemoattractant protein-1 (MCP-1) expression, in a mechanism that potentially involves TGFβ1 (54). MCP1 plays a similar role in other tumors (55), but whether PI3K–AKT induced MCP1 expression can be generalized to other tumors remains to be investigated. mTORC2 appears to be particularly important for differentiation of M2 macrophages (as opposed to M1), as not only monocytes recruitment but also monocyte polarization is involved in tumor progression (56); therefore, mTORC2 plays a dual immunosuppressive role.

Antigen-presenting cells (APCs), especially dendritic cells (DCs), are crucial in mounting antitumor immune response (57). Indeed, abrogation of mTORC2 signaling in the professional APCs, DCs, led to enhanced tumor eradication possibly *via* engagement of CTLs (58). Rapamycin administration augmented the expression of costimulatory molecules and enhanced DC life span, *via* modulation of glucose metabolism (59). These data suggest that mTOR signaling in APC cells imposes an immunosuppressive environment.

## Myeloid-Derived Suppressor Cells (MDSCs)

Myeloid-derived suppressor cells are a heterogeneous population defined as CD11b + Gr1 + cells. Based on Ly6G and Ly6C expression, MDSCs can be further classified as granulocytic or monocytic subsets, respectively. Both CD11b + Ly6G + and CD11b + Ly6C + cells play immunosuppressive roles. The allosteric mTORC1 inhibitor, rapamycin, inhibits MDSC accumulation in tumors and skin allografts (60). In breast cancer, accumulation of MDSCs in tumors occurred *via* G-CSF. Rapamycin administration or *Raptor* deletion (a core-component of mTORC1) reduced G-CSF levels (61), suggesting that mTORC1 in tumor cells attracts MDSCs by upregulating G-CSF. Increased G-CSF levels also correlated with elevated mTOR activity in human tumors. Interestingly, there is correlation between presence of TICs, elevated mTORC1 signaling, and G-CSF production. Moreover, rapamycin administration leads to reduced TIC levels (61). These data suggest that mTOR activity in a subset of cells within the tumor mass (i.e., intra-tumoral heterogeneity) mediates MDSC accumulation.

## Other Cells of the Tumor Microenvironment: CAFs

Fibroblasts are not only involved in the deposition of stromal extra-cellular matrix (ECM) but also in the secretion of growth factors. CAFs seem to play a role in cancer progression and initiation, particularly in stroma-rich tumors like pancreatic cancers (62, 63). In pancreatic tumors, CAFs are also involved in resistance to anticancer drugs (64). Interleukin-6 (IL-6) is linked to resistance-to-cancer drug therapies (65), possibly *via* its downstream effector pSTAT3 (66). In pancreatic CAFs, the somatostatin receptor sst1 inhibits mTOR-mediated IL-6 protein synthesis, thereby counteracting mTOR/IL-6-driven resistance to anticancer drugs (67). How mTOR regulates IL-6 expression in stromal cells remains to be investigated, but this mechanism seems to involve the quintessential mTORC1 target, 4E-BP1 (67). In lung carcinoma, paracrine IGF-II secretion by CAFs activated insulin growth factor receptor 1 (IGF1R) signaling in cancer cells, possibly activating a TICs (stemness)-like phenotype (68). Conversely, in irradiated tumors, IGF-II secreted from CAFs appears to block mTORC1 signaling in neighboring cancer cells. mTORC1 inhibition allowed autophagy initiation and thereby tumor regrowth (69). It seems counterintuitive that mTOR inhibition allows tumor growth, but possibly under stress or nutrient-poor conditions autophagy initiation provide the required nutrients. Nevertheless, this hypothesis needs to be examined in other cancer models. Yet, liver specific *Raptor* knockout mice (abrogated mTORC1 signaling) developed more HCC when challenged with the hepato-carcinogen diethyl-nitrosamine, as compared with wild-type mice (70). These data suggest that “too low” mTORC1 activity may also be oncogenic. Taken together, it is likely that the response to drug therapies is not only dependent on stromal cells and their secretome but also on the conditions in which therapies are given.

## mTOR on the Receiving End of Cancer Immunity

mTOR signaling is also on the receiving end of cues coming from the tumor microenvironment. For example, non-tumorigenic (stromal) cells of the tumor microenvironment secrete MCP1 to activate the mTOR pathway in neighboring breast cancer cells (71). Moreover, metabolic activation of natural killer (NK) cells is dependent on IL-15 stimulation to prompt intracellular mTOR signaling (72). NK cells are suggested to play a pivotal role in cancer control and are increased in metastatic melanoma (73). Conversely, TGF-β represses mTOR signaling, both in mice and humans, to inhibit NK cell activation (74), suggesting an mTOR-dependent immune suppressive role for TGF-β in tumor microenvironment. Additionally, genetic activation of mTORC1 (mutated TSC) causes impairment of NK cell development (75). Notably, mTOR also regulates Th1 and Th2 differentiation; and while mTORC1 is distinctly critical for Th1 and Th17 differentiation, mTORC2 seems to promote Th2 differentiation (76). Furthermore, mTORC1 regulates CD8 + T-cell effector function (77), thereby allowing better clearance of tumor cells. Although mTORC2 seems to be dispensable for the effector function of CD8 + T cells, it is critical for generation of CD8 + memory cells (77). Further studies are required to examine how extracellular signals affect mTOR in T cells; nonetheless, the data demonstrate that mTOR signaling differentially regulates T cells.

## Clinical Implications

**Figure 2:**
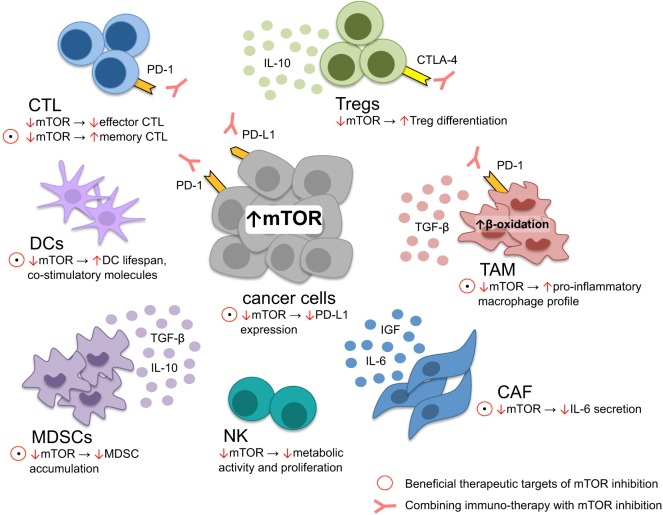
Tumor microenvironment modulation through mTOR. mTOR inhibition induces memory cytotoxic CD8 + T lymphocytes (CTL) formation while reducing effector CTL function, critical for cellular antitumor response. In dendritic cells, lifespan and the expression of costimulatory molecules is increased upon mTOR suppression, leading to improved foreign-antigen recognition. On the other hand, metabolic NK-cell function, essential for antitumor response, is diminished upon mTOR inhibition. Myeloid derived stem cells (MDSC), regulatory T cells (Tregs), tumor-associated macrophages (TAM), and cancer-associated fibroblasts (CAF) contribute to tumor immune-evasion and tumor growth. The immune-suppressive environment generated through MDSC is limited through mTOR blockage by restraining MDSC accumulation. Similarly, anti-inflammatory TAM may be skewed toward a more pro-inflammatory profile upon mTOR inhibition. CAF secrete various cytokines promoting tumor growth and therapy resistance, counteracted by mTOR blockage. In contrast, Tregs are preferentially differentiated upon mTOR downregulation. Within the majority of cancer cells, the PI3K–mTOR–AKT pathway is upregulated, driving PD-L1 expression maintaining an immune-suppressive state within the tumor microenvironment: a process that may be interrupted through mTOR inhibition. However, not all therapeutic targets of mTOR inhibition seem to be beneficial, such as reducing effector CTL function and T-Reg differentiation. Accordingly, rationale exists to combine anti-PD-1/PD-L1 or anti-CTLA-4 and mTOR inhibitors, alleviating reduced CTL effector function and Treg differentiation.

Various mTOR inhibitors are in ongoing clinical trials and the FDA-approved rapalog everolimus is used in various cancer cell types (10). Because mTOR signaling plays a key role in cancer and immune cell function (78), it is possible that some of the anticancer effect of mTOR inhibitors is *via* immune modulation (Figure 2). Indeed, rapamycin is clinically used for prevention of renal graft rejection and is traditionally considered as a “pure” immunosuppressant, possibly by blocking T-cell activation. However, as discussed above, mTOR seems to play a more complex role in immunity. Under certain conditions, mTOR inhibition poses an immune-activating function, such as induction of memory CD8 + T cell (77) that may in turn increase the durability of antitumor effector T-cell function. It is also likely that ATP kinase mTOR inhibitors, blocking robustly mTORC1, and mTORC2 signaling (79) act on cancer cells, as well as on the tumor microenvironment.

While checkpoint and mTOR inhibitors have revolutionized cancer treatment, as monotherapies these drugs seem to be insufficient to fully block cancer progression. Oncogenic PI3K–mTOR–AKT pathway reduces T-cell tumor infiltration and causes inferior outcome after PD-1 inhibition (31), providing a rationale for the design of combination therapies of mTOR and immune checkpoint inhibitors, as recently shown for HCC (80) (Figure 2). Nonetheless, the specific oncogenic mechanism downstream of mTOR remains unknown. Understanding these pathways is critical for the rational design of selective inhibitors. The combination of checkpoint and mTOR inhibitors might be limited by its side effects because: (i) PI3K–mTOR–AKT signaling plays a critical role in physiological cell homeostasis, (ii) rapamycin administration reduces the effector CD8 + T-cell function (that are otherwise required for execution of anticancer effect), and (iii) possibly relieve negative feedback loops that may induce compensatory pathway activation.

## Conclusion

Collectively, the above suggests that mTOR signaling has both tumor-intrinsic and tumor-extrinsic (i.e., tumor microenvironment) activities. mTOR-kinase quickly responds to stimuli in the tumor microenvironment and executes various (possibly opposing) effects on immune cells. Thus, a prime challenge is to dissect the role of mTOR in the different cell types in the tumor microenvironment and to assess the overall “net effect” of mTOR blockade.

## Author Contributions

YG wrote the original draft. YG, TMN, and JR wrote and approved the final version of the review.

## Conflict of Interest Statement

The authors declare that the research was conducted in the absence of any commercial or financial relationships that could be construed as a potential conflict of interest.
